# Exploring the Multidimensional Links Between Trait Mindfulness and Trait Empathy

**DOI:** 10.3389/fpsyt.2021.498614

**Published:** 2021-08-04

**Authors:** Toshiyuki Himichi, Hidekazu Osanai, Takayuki Goto, Hiroyo Fujita, Yuta Kawamura, Adam Smith, Michio Nomura

**Affiliations:** ^1^School of Economics & Management, Kochi University of Technology, Kochi, Japan; ^2^Faculty of Modern Communication Studies, Hamamatsu Gakuin University, Hamamatsu, Japan; ^3^School of Human Cultures, The University of Shiga Prefecture, Hikone, Japan; ^4^Graduate School of Education, Kyoto University, Kyoto, Japan; ^5^Graduate School of Humanities, Kobe University, Kobe, Japan; ^6^Japan Society for the Promotion of Science, Tokyo, Japan; ^7^Graduate School of International Development, Nagoya University, Nagoya, Japan

**Keywords:** empathy, mindfulness, effortful control, attention, emotion regulation, alexithymia

## Abstract

Empathy and mindfulness are currently major topics of scientific interest. Although it is well-known that mindfulness—typically as an outcome related to meditation—generates empathy at the state level, only a small number of studies have documented the trait (i.e., personality) level association between mindfulness and empathy. Furthermore, the underlying mechanisms (subcomponents and mediator variables) that support this association remain unclear. Thus, here, with a focus on the trait level, we investigated relationships among multiple subcomponents of trait mindfulness and trait empathy (Study 1). Next, we reexamined the aforementioned relationships in an independent sample, with the further aim of investigating relevant mediation factors (Study 2). We found that two attention-related components of trait mindfulness—observing and acting with awareness—reliably and positively related to both affective and cognitive dimensions of trait empathy (i.e., empathic concern and perspective taking). Furthermore, we found that effortful control, reappraisal, and trait alexithymia mediated relationships between the aforementioned attention-related components of trait mindfulness and empathic concern. Taken together, our results suggest that the links between mindfulness and empathy are multidimensional and complex. These findings may ultimately contribute to an understanding of the mechanisms underlying the positive effects of meditation on empathy.

## Introduction

The capacity for empathy appears to be indispensable to human sociality; it not only serves as a window into the emotional and cognitive states of others (1, 2) but also motivates altruistic behavior directed toward those in need (3). A number of studies have shown that trait empathy positively correlates with well-being (4–6), and thus it is conceivable that the promotion of empathy may contribute to the enhancement of people's lives. To this end, a large number of researchers have focused on meditation as a potential means for the promotion of empathy [e.g., (6)].

Researchers have shown that practicing various forms of meditation—ranging from loving-kindness meditation to focused attention—leads to increases in self-reported empathy (7, 8) and related behaviors such as prosociality (9). Furthermore, meditation is reliably associated with empathy-related brain activity (10–12). A recent meta-analysis likewise supports the relationship between meditation and empathy (13). The authors investigated the effects of a wide range of methods of meditation training (and mindfulness-related activities) on prosocial behavior and self-reported empathy. With effect sizes ranging from small to medium, Luberto et al. (13) confirmed the effectiveness of meditation as a means for the promotion of empathy.

Although many researchers have documented the positive effects of meditation (and resultant mindfulness) on empathy, the relationship between mindfulness and empathy at the trait or personality level remains relatively unclear. To better understand the relationship between trait mindfulness and trait empathy in detail, one effective approach is to investigate relationships between these variables' underlying components (14, 15). A growing body of research shows that both trait mindfulness (16) and trait empathy (17) appear to be constructed by multidimensional subcomponents. Therefore, it is possible that the strength of the relationships between trait mindfulness and trait empathy may differ at the subcomponent level. The present study investigated the multiple correlational relationships that exist between the subcomponents of trait mindfulness and trait empathy. Investigating differences in the relationships between the trait-level subcomponents of mindfulness and empathy may contribute to a present understanding of what facets of a mindful personality predict the tendency to be empathetic toward other people. Moreover, if process X is important for behavior Y, it can be considered that intervention for X is effective for enhancing Y. Therefore, investigating relationships between subcomponents of mindfulness and empathy might contribute to a future understanding of how meditation facilitates empathy (e.g., what cognitive/affective processes mediate the relationship between meditation and empathy) and predict what type of meditation is more effective for interventions designed to increase empathy.

As a multidimensional approach to trait empathy, two concepts are considered “primary” components of empathy, as they map onto the other-oriented constructs of emotional empathy (i.e., *empathic concern*) and cognitive empathy (i.e., *perspective taking*) (18, 19). *Empathic concern* represents the degree to which one feels other-oriented kindness, whereas *perspective taking* represents the degree to which one takes the perspective of others. These components are measured by the Interpersonal Reactivity Index [IRI; (20)].

A multidimensional approach has also been applied to trait mindfulness. Baer et al. (16) suggested a model of mindfulness comprising five subcomponents: *observing* represents the degree to which one pays attention to internal (e.g., emotional) and external (e.g., sensational) experiences; *describing/labeling* represents the degree to which one expresses his/her own emotions in thoughts and words; *acting with awareness* represents the degree to which one focuses on one's own present-moment experience; *non-judging* represents how much one refrains from judgments about one's own negative thoughts and feelings; and *non-reactivity* represents how much one controls reactions to one's own negative emotions. Previous studies have shown that various subcomponents of mindfulness [measured by the Five Facet Mindfulness Questionnaire [FFMQ]; (16)] tend to be increased by various methods of meditation training (21–24). Furthermore, several correlational studies have shown that almost all subcomponents correlate positively with cognitive functioning (e.g., emotion regulation, effortful control) and positive psychological tendencies (e.g., psychological well-being, emotional intelligence) and correlate negatively with negative psychological tendencies, such as depression and alexithymia (16, 21, 22, 25).

Previous studies have investigated the multidimensional relationships between the subcomponents of trait mindfulness and trait empathy. Keane (26), for example investigated how mindfulness practice affected a sample of psychotherapists and analyzed correlations between subcomponents of the FFMQ and the IRI. Keane found that all subcomponents of the FFMQ positively and significantly correlated with *perspective taking* and that *observing* positively and significantly correlated with *empathic concern*. Additionally, in this study, a*cting with awareness, non-judgment*, and *non-reactivity* correlated positively with *empathic concern* with medium—but statistically nonsignificant—effect sizes (*r*s > 0.26). Although these findings are important to understand the relationship between mindfulness and empathy, there are limitations to the robustness of these findings due to the relatively small sample size (*n* = 40). Another study (15) investigated correlations between the FFMQ and the Questionnaire of Cognitive and Affective Empathy (QCAE) which assesses the cognitive component (i.e., the reading of others' minds) and emotional component (i.e., vicarious emotional responses) of empathy (27). This study found that cognitive empathy correlates positively with almost all subcomponents of the FFMQ, whereas emotional empathy correlates negatively with almost all subcomponents of the FFMQ. More recently, Fuochi and Voci (14) investigated the relationship between components of trait mindfulness (measured by the FFMQ) and trait empathy (measured by the IRI) using multiple linear regression analysis. Consistent with the previous studies described above (15, 26), they have shown that all mindfulness components repeatedly and positively correlated with *perspective taking*, except *non-judging*. However, their results were partly inconsistent with the two abovementioned studies. They have also shown that *observing* and *acting with awareness* repeatedly and positively correlated with *empathic concern*, whereas *non-reactivity* repeatedly and negatively correlated with *empathic concern*. Therefore, evidence of relationships between trait mindfulness and emotional empathy is still not convergent. Thus, more studies are necessary to clarify the multidimensional nature of the connection(s) between mindfulness and empathy.

The present studies aimed at investigating the multifaceted relationships between various subcomponents of trait mindfulness and trait empathy using path analysis to investigate the relationship between multiple variables. Because all of our variables are observed (as opposed to latent) and the relationships between predictors, mediators, and dependent variables are assumed to be unidirectional, we used path analysis, instead of Structural Equation Modeling, to analyze the results of Studies 1 and 2. Likewise, we produced path models to visualize the results of both studies.

## Study 1

We investigated relationships between each subcomponent of the FFMQ and two subcomponents of the IRI: *empathic concern* and *perspective taking*, in a Japanese sample. Based on findings by Keane (26) which measured empathy with the IRI, we predicted that all subcomponents of the FFMQ would correlate positively with *empathic concern* and *perspective taking*. In the findings by Keane, all subcomponents of FFMQ positively and significantly correlate with *perspective taking*, whereas *empathic concern* showed positive, medium-sized, but nonsignificant correlations with four components of trait mindfulness (i.e., *non-judgment, non-reactivity, describing/labeling*, and *acting with awareness*). Because the sample size of these analyses was small, Keane's study might not have had sufficient power. Therefore, we predicted that the positive correlations with *empathic concern* might become significant under a sufficient sample size. Note that the correlation between *empathic concern* and *describing/labeling* in Keane was small and non-significant. However, recall that the *describing/labeling* component of trait mindfulness pertains to the ability to express and understand one's own emotions, and *empathic concern* is the central emotional component of empathy. Therefore, because *describing/labeling* and *empathic concern* are both emotional components of each trait, we hypothesized that these variables might be positively correlated.

### Materials and Methods

#### Participants

Four hundred sixteen Japanese adults (208 men and 208 women; mean age = 39.41 years, *SD* = 11.19, range = 20–59 years) who were registered with a research company (MACROMILL, Inc.) participated in this study *via* an online survey. The research company managed compensation *via* tokens that participants could exchange for goods or cash.

Participants had various occupations (e.g., public service worker, family-operated business worker, and student). Although we did not measure meditation experience (e.g., length/amount of meditation experience) in the survey, we consider that most participants were likely naive to meditation; this is because we used a similar community sample in Study 2 in which we found this to be the case (see the Participants section in Study 2 for more details). We informed the participants about the nature (in terms of requirements and content) of this study before responding. After participants consented, they began responding to the survey. The procedure of the present study was retrospectively reviewed and approved by an ethical committee of the Department of Cognitive Psychology in Education from Kyoto University.

#### Procedure

The participants completed the IRI and FFMQ. Although the participants completed several other noninvasive, personality-related questionnaires for purposes unrelated to the present study, we do not report details and their results here. Participants completed the IRI first, and the order of the remaining questionnaires was randomized. The item order within each questionnaire was also randomized. The survey required participants to respond to each item before proceeding. Thus, the present data have no missing values. However, participants were free to stop responding at any point during the survey.

#### Measures

The FFMQ includes 39 items and is rated on a five-point scale [1: *never or rarely agree*; 5: *always or often agree*; (16); Japanese version: (25)]. The FFMQ consists of five subcomponents: *observing* (eight items), *non-reactivity* (seven items), *non-judgment* (eight items), *describing/labeling* (eight items), and *acting with awareness* (eight items). Internal consistencies of these sub-scales showed no serious problems (Table 1; αs > 0.73).

**Table 1:** Basic statistical values, internal reliabilities, and correlations of each scale in Study 1.

	**Scale**		***M***	***SD***	**α**	**1**	**2**	**3**	**4**	**5**	**6**	**7**	**8**
1	IRI	Personal distress	3.14	0.64	0.78	–							
2		Empathic concern	3.31	0.54	0.72	0.14^**^	–						
3		Perspective taking^a^	3.04	0.63	0.71	0.11^*^	0.35^***^	–					
4		Fantasy scale	3.13	0.65	0.76	0.25^***^	0.34^***^	0.27^***^	–				
5	FFMQ	Observing	2.83	0.63	0.80	0.05	0.23^***^	0.44^***^	0.24^***^	–			
6		Non-reactivity	2.82	0.55	0.73	−0.27^***^	0.09^†^	0.30^***^	0.00	0.49^***^	–		
7		Non-judging	3.14	0.58	0.81	−0.34^***^	−0.15^**^	−0.36^***^	−0.25^***^	−0.47^***^	−0.31^***^	–	
8		Describing/Labeling	2.83	0.66	0.84	−0.40^***^	0.22^***^	0.17^***^	0.10^*^	0.36^***^	0.35^***^	0.05	–
9		Acting with awareness	3.35	0.64	0.85	−0.36^***^	0.07	−0.11^*^	−0.14^**^	−0.30^***^	−0.14^**^	0.56^***^	0.18^***^

a *Two items from perspective taking were excluded. IRI, Interpersonal Reactivity Index; FFMQ, Five Facets Mindfulness Questionnaire;*

*** *p < 0.001;*

** *p < 0.01;*

* *p < 0.05;*

† *p < 0.10*.

The IRI includes 28 items and is rated on a five-point scale [1: *strongly disagree*; 5: *strongly agree*; (20); Japanese version: (28)]. All subscales consist of seven items. Internal consistencies of these subscales (αs > 0.72; see Table 1) showed no serious problem except for *perspective taking* which was only moderate (α = 0.61). To improve the internal consistency of *perspective taking*, two items (#3 and #15) for which the total-item correlation was relatively low (*r*s < 0.42) were excluded from analysis (α = 0.71; Table 1). Note that in a previous study using a Japanese sample, internal consistency was also improved after excluding the same two items (28). Data from the IRI were used in a previous, unrelated study, which focused on scale development (28).

#### Data Analyses

**Figure 1:**
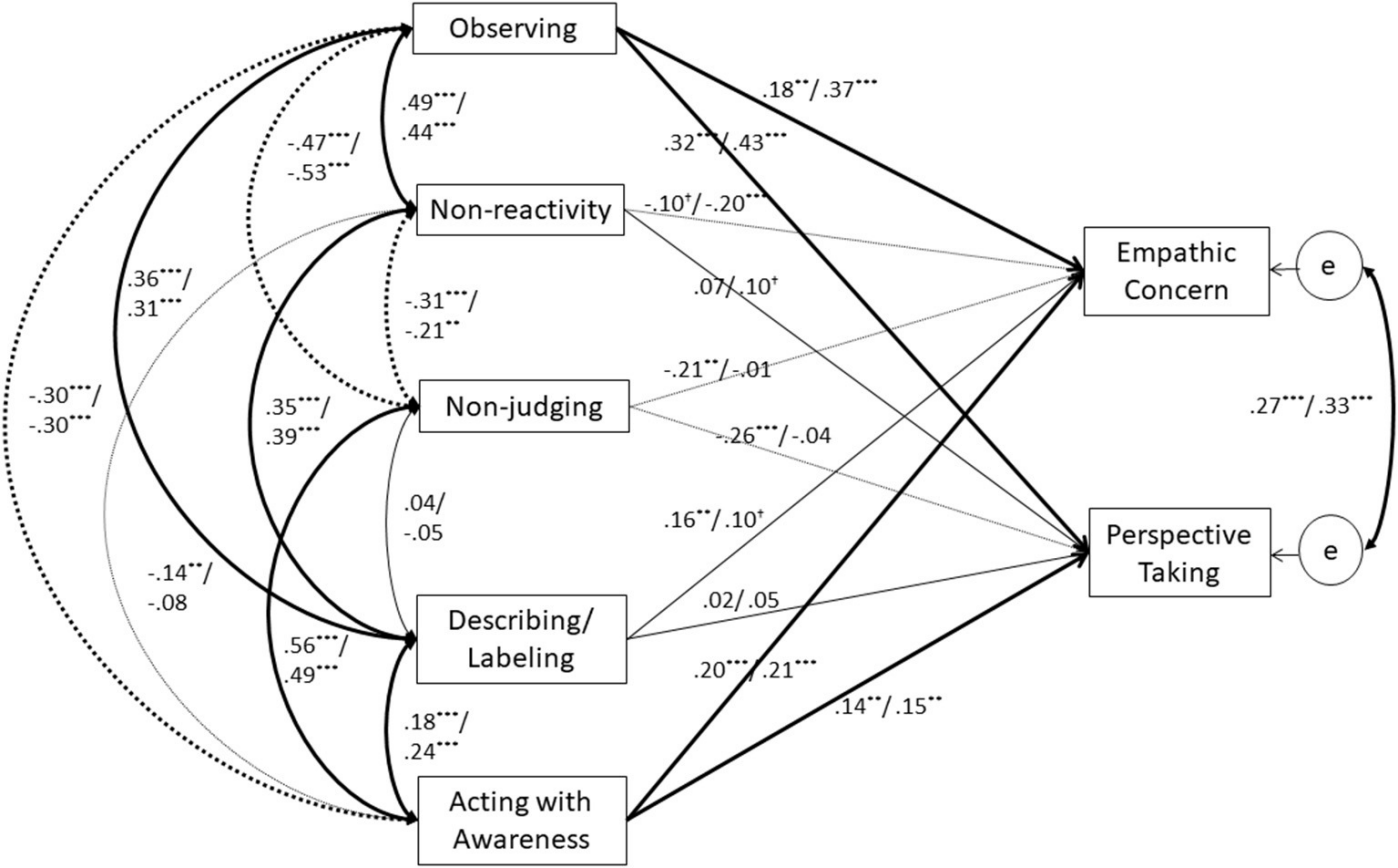
Results of path models for Studies 1 and 2 regarding effects of five facets of mindfulness on empathic concern and perspective taking. Each value indicates a correlation (*r*) or standardized coefficient (β). Left values are results of the Study 1, and right values are results of Study 2. Weights of paths correspond to statistical significance of each path. Continuous lines indicate positive relations, and dotted lines indicate negative relations. ***p < 0.001; **p < 0.01; ^†^*p* < 0.10; e, error.

Means, standard deviations, and Cronbach's alphas of each scale, and their correlations were analyzed using the psych package [ver. 1.6.4; (29)] for R [ver.3.3.0; (30)]. Furthermore, we conducted path analyses *via* Mplus [ver. 7.4; (31)]. This allowed us to estimate the direct effects of each subscale of the FFMQ on the *empathic concern* and *perspective takin*g subcomponents of the IRI. The average of each scale was used to estimate the model. We included all correlations and paths in the estimated model; thus, the model is a saturated model (see Figure 1).

To investigate possible effects of demographic variables, we tested another model using path analysis. We entered age and a dummy variable of gender (1: men: 2: women) as independent variables in the abovementioned model. Beadle and De La Vega (32) reviewed studies investigating age differences in trait empathy and suggested that aging (sometimes) influences *perspective taking*, but not *empathic concern*. Furthermore, previous studies showed that women typically have higher trait empathy than men (20, 33–35). Nevertheless, results from our analyses with age and gender were the same as the main results of path analysis (see Supplementary Material).

### Results

Correlations are summarized in Table 1. The direction of correlations between the FFMQ and IRI varied among subcomponents. Regarding internal correlations among the FFMQ, *observing* negatively and significantly correlated with *non-judging* and *acting with awareness* (*r*s < −0.30, *p* < 0.001). *Non-reactivity* correlated negatively and significantly with *non-judging* and *acting with awareness* (*r*s < −0.14, *p* < 0.003). Other internal correlations among the FFMQ were significant and positive (*r*s > 0.18, *p* < 0.001), except a nonsignificant correlation between *non-judging* and *describing/labeling* (*r* = 0.05, *p* = 0.365).

Results of path analysis are illustrated in Figure 1. *Observing* and *acting with awareness* significantly and positively correlated with both *empathic concern* and *perspective taking* (βs > 0.13, *p* < 0.009). *Describing/labeling* significantly and positively correlated with only *empathic concern* (β = 0.16, *p* = 0.003). However, the model revealed that effects of the FFMQ on the IRI are not only positive. Specifically, *non-judging* significantly and negatively correlated with *empathic concern* and *perspective taking* (βs < −0.21, *p* < 0.002). Finally, there were no significant relationships between *non-reactivity* and the two focal subcomponents of empathy (|β|s < 0.10, *p* > 0.083).

### Discussion

We investigated relationships between the subcomponents of trait mindfulness and trait empathy in Study 1 utilizing a large community-based sample. In this discussion, we focus on the results of our path model, starting out with an exploration of positive relationships. Path analysis revealed that *empathic concern* and *perspective taking* positively correlated with *observing* and *acting with awareness*. These relationships were found in previous studies using the FFMQ and IRI (14, 26). In a previous study that used the QCAE for measuring trait empathy, only the relationship between *acting with awareness* and emotional empathy was different [i.e., a negative relationship; (15)]. Emotional empathy measured by the QCAE uses vicarious emotional responses (27), whereas emotional empathy measured by the IRI uses compassion for a person in a distressful situation (20). Therefore, differences in the measured components of emotional empathy may cause inconsistency in findings.

Our results also revealed that *describing/labeling*—the subcomponent of the FFMQ that measures differences in the ability to express thoughts and emotions—positively correlated with *empathic concern*, but did not correlate with *perspective taking*. Previous studies have found that *describing/labeling* positively correlated with cognitive empathy, but did not correlate with emotional empathy (14, 15, 26). The relationship between *describing/labeling* and emotional empathy found in the present study is inconsistent with these previous studies. Furthermore, our model did reveal a few negative relationships, specifically when it comes to *non-reactivity* and *non-judging*. The *non-judging* component of trait mindfulness negatively correlated with both *empathic concern* and *perspective taking*. Fuochi and Voci (14) have found that in multiple-regression analysis, *non-judging* did not correlate with *perspective taking* across three studies and did not relate to *empathic concern* in two of three studies. Another previous study has reported that *non-judging* positively correlated with *empathic concern* and *perspective taking* (26). Finally, MacDonald and Price (15) reported that *non-judging* negatively correlated with emotional empathy but did not significantly correlate with cognitive empathy. Therefore, the robustness of the negative relationships revealed by Study 1's path model is unclear.

As described above, there are several inconsistencies between the results of Study 1 and previous studies (14, 15, 26). Perhaps moderating variables such as culture (previous studies used Western samples; the present study used an East Asian sample) may be one reason for these inconsistencies. For example, in the existing literature, there are some differences in the internal correlations among subcomponents of the FFMQ between the results from Western participants and Eastern participants. Specifically, *observing* has been documented as negatively correlating with *non-judging* and *acting with awareness* in Eastern cultures (25, 36–41), and our results are consistent with these results. In contrast, in Western cultures (14–16, 22), almost all components of the FFMQ positively correlated with each other. Considering that the factor structure of the Japanese version of the FFMQ was developed as a replication of the popular English version of the scale—without an effort to (re)integrate Japanese/Buddhist conceptions of mindfulness (25)—the meaning of observed psychological traits may differ slightly by culture. Grossman (42), for example pointed out that constructs of mindfulness used by Western psychologists often remove Buddhist elements such as altruistic (i.e., religious) motivation and tend to extend the construct beyond the meditative context. Such fundamental cultural differences in approaches to mindfulness may influence the relationship between trait mindfulness and trait empathy.

Another possible account for cultural differences in the relationships between trait mindfulness and empathy might be interoception. For example, cultural differences were found in the *describing/labeling* scale: This scale positively related to *perspective taking* in previous, Western, studies (14, 15, 26), but this scale positively related to *empathic concern* in the present, East-Asian, study. There are documented East-West cultural differences in interoception (43). Western people are higher in the *accuracy* of interoception than Eastern people (44). Accurate interoception facilitates the self-other distinction *via* effects on body ownership and representation of the self (45). The self-other distinction is one important process for *perspective taking* [e.g., (46)], and thus, this might help explain why *describing/labeling* has a positive relationship to *perspective taking* in Western cultures. By contrast, people in Eastern cultures use somatic words more frequently during speech about emotional experiences than people in Western cultures (47, 48); thus, bodily sensation is relatively more strongly related to emotional experiences in Eastern cultures. In sum, *describing/labeling* might have a more positive relationship to feeling warm emotions toward others (i.e., *empathic concern*) in Eastern cultures, whereas *describing/labeling* may have a more positive relationship with accurately understanding others (i.e., *perspective taking*) in Western cultures. Note, however, that interpretations of these differences in interoception are speculative, and these accounts should be treated carefully.

Taken together, the results show that subcomponent relationships between trait mindfulness and trait empathy are diverse and complex. As described above, due to the inconsistency of our results with previous findings (14, 15, 26), next we examine whether Study 1 can be replicated. Furthermore, we investigate possible mediation factors. Thus, we conducted Study 2 to replicate and extend Study 1.

## Study 2

In Study 2, we investigated whether the relationships between mindfulness and empathy at the trait level found in Study 1 can be replicated using an independent dataset. Furthermore, we investigated several potential mediating variables between trait mindfulness and trait empathy.

A meta-analysis has shown that meditation training enhances not only empathy but also a wide variety of psychological traits and physiological outcomes including improvements in emotional regulation and executive functioning (49). Such cognitive and affective enhancements may relate to a general positive effect of meditation on empathy. Hölzel et al. (50) reviewed literature about empirical effects of mindfulness meditation and suggested that meditation produces four specific cognitive and affective changes—improvements in attention regulation, body awareness, emotion regulation, and changes in self-perspective. Attentional control and body awareness are also important for empathy (50, 51), and emotion regulation is one of the moderating processes of emotional empathic responses (51). Therefore, we predicted that three traits (emotion regulation, effortful control, and alexithymia), which likely relate to meditation and empathy, may act as mediation factors between trait mindfulness and trait empathy. Although mediation effects of alexithymia and emotion regulation in relationships between mindfulness and empathy have been investigated in previous studies [alexithymia: (15); emotion regulation: (14)], effects of these variables have not been investigated simultaneously. Furthermore, even though changes in the perspective of the self may be an important mediation factor, the present study did not include it due to difficulty quantifying the measurement of this trait (50).

Attentional control, which is the ability to focus and disengage attention intentionally, is one of the central processes in meditation. Specifically, meditation generally trains the focusing and detaching of attention (mindfulness meditation) or the spreading of compassion from self to others (loving-kindness meditation), which also involves attentional control (50). Attentional control appears to play an important role for emotion regulation (52). Moreover, Engen and Singer (53) have investigated how emotion regulation strategies based on compassion-meditation and reappraisal (i.e., the voluntary changing of appraisals for emotional stimuli) relate to subjective or neural empathic responses, in the meditation experience of participants. The researchers have shown that both emotion regulation processes involved in both compassion meditation and reappraisal decreased subjective negative emotions and increased subjective positive emotions after observing clips that contained people in distressed situations. A related study has likewise shown that compassion training decreased negative emotions and increased positive emotions (54). Positive emotions in these studies are other-oriented warm emotions (e.g., love and affiliation) referred to as *empathic concern*. Therefore, these studies suggest that emotion regulation may partially mediate the positive relationship between trait mindfulness and trait empathy (specifically, *empathic concern*).

Second, cognitive inference of others' minds, so-called “Theory of Mind” or “mentalizing” (55), which is conceptually similar to cognitive empathy (1) is partly supported by effortful control such as executive functioning (56). People have an egocentric bias which overestimates the degree to which self-knowledge and self-perspective information are useful for inferring the states of others' minds (46). Of course, because one's own and others' knowledge, beliefs, and thoughts are different, inhibition of self-knowledge and self-perspective information is important for appropriate inference into others' mental states. Cognitive psychological studies have shown that high cognitive load interferes with performance on theory of mind tasks (57–60). These findings imply that increased effortful control—which may be achieved *via* mindfulness meditation training [see (49), for a meta-analytic review]—should positively affect cognitive empathy. Therefore, effortful control may partially mediate the positive relationship between trait mindfulness and trait level differences in empathy as measured in terms of *perspective taking*.

In addition to emotion regulation and effortful control, we thirdly predicted that alexithymia may be an important mediating factor. Previous studies have shown that alexithymia (i.e., difficulty expressing and understanding one's emotional state) negatively correlates with almost all subcomponents of trait mindfulness (16, 22, 25). Neuroimaging findings furthermore show that people high in alexithymia have decreased activation in the anterior insula while observing other people's pain (61). These results suggest that high trait alexithymia is associated with a decrease in sharing other people's pain, because the insula is a major neural basis of empathy (62, 63). By contrast, a meta-analysis of neuroimaging research on meditation training showed that insula activity is increased by many common kinds of meditation methods [i.e., focused attention, open monitoring, and loving-kindness; (64)]. These results imply that meditation training enhances emotional empathy through the reduction of alexithymia tendencies. Hölzel et al. (50) predicted that body awareness, an important outcome of meditation, is associated with empathy, because understanding one's own physical and emotional states is important for understanding and sharing other's physical and emotional states. Several studies have shown that alexithymia is associated with decreased body awareness using various kinds of physiological measurements (65–67). These lines of evidence support our predictions that trait alexithymia mediates the relationships between trait mindfulness and trait empathy. However, inconsistent with these findings, MacDonald and Price (15) have shown that alexithymia partially mediates the relationship between trait mindfulness (*describing/labeling*, and *acting with awareness*) and cognitive empathy (measured by the QCAE), but *not* emotional empathy. Taken together, although the relationships predicted by previous findings are somewhat inconsistent, previous studies suggest that alexithymia is a potentially important mediator between trait mindfulness and trait empathy.

### Materials and Methods

#### Participants

Five hundred sixteen Japanese adults (258 men and 258 women; mean age = 39.45 years, *SD* = 11.10, range = 20–59 years) participated in this study. This study was also a web-based survey, and the participants were recruited from a research company (MACROMILL, Inc.). As with Study 1, the research company managed compensation *via* tokens that participants could exchange for goods or cash. None of the participants in Study 2 took part in Study 1.

We assayed for participants' previous experience of meditation practice (see Supplementary Table 1). Almost all participants (*n* = 468) had never practiced meditation. Of the remaining participants (*n* = 48; mean practice time = 25.06 h, *SD* = 44.40, range 0–245 h), those who had practiced meditation for longer than 28 h [the equivalent of longer than 30 min per day for 8 weeks, which is a time period often used in intervention studies; e.g., (7)] were rare (*n* = 10). Therefore, we considered that the participants were generally naive in terms of meditation practice, and utilized data from all participants. We informed the participants about the nature (in terms of requirements and content) of this study before responding. After participants consented, they started responding to the survey. The procedure of the present study was retrospectively reviewed and approved by an ethical committee of the Department of Cognitive Psychology in Education from Kyoto University.

#### Procedure

This study was conducted by the same research company as with Study 1 (MACROMILL, Inc.), and its procedure was similar to Study 1. The participants responded to five questionnaires followed by several questions about meditation experience. The participants also completed an unrelated questionnaire, the details, and results of which we do not report in the present manuscript. The order of response to the six questionnaires and items of each questionnaire was randomized.

#### Measures

Scales used to measure empathy and mindfulness were the same as with Study 1 (IRI and FFMQ). Their internal consistencies were relatively good (Table 2; αs > 0.74) except for *perspective taking* which was moderate (α = 0.66). Two items (#3 and #15) were also excluded from analyses as in Study 1, and the internal consistency of *perspective taking* was improved (α = 0.75). Data from the IRI were used in a previous, unrelated study, which focused on scale development (28).

**Table 2:** Basic statistical values, internal reliabilities, and correlations of each scale in Study 2.

	**Scale**		***M***	***SD***	**α**	**1**	**2**	**3**	**4**	**5**	**6**	**7**	**8**	**9**	**10**	**11**	**12**
1	IRI	Personal distress	3.07	0.62	0.74	–											
2	IRI	Empathic concern	3.32	0.59	0.76	0.06	–										
3	IRI	Perspective taking^a^	3.03	0.66	0.75	−0.01	0.40^***^	–									
4	IRI	Fantasy scale	3.13	0.68	0.79	0.23^***^	0.30^***^	0.29^***^	−								
5	FFMQ	Observing	2.84	0.64	0.79	0.04	0.25^***^	0.47^***^	0.29^***^	–							
6	FFMQ	Non-reactivity	2.84	0.59	0.75	−0.33^***^	−0.01	0.30^***^	−0.01	0.44^***^	−						
7	FFMQ	Non-judging	3.16	0.61	0.82	−0.25^***^	−0.06	−0.22^***^	−0.25^***^	−0.53^***^	−0.21^***^	–					
8	FFMQ	Describing/Labeling	2.85	0.66	0.83	−0.35^***^	0.19^***^	0.26^***^	0.01	0.31^***^	0.39^***^	−0.05	−				
9	FFMQ	Acting with awareness	3.32	0.66	0.85	−0.38^***^	0.14^**^	0.01	−0.19^***^	−0.30^***^	−0.08^†^	0.49^***^	0.24^***^	–			
10	ERQ	Reappraisal	4.17	0.89	0.84	−0.11^*^	0.16^***^	0.41^***^	0.13^**^	0.36^***^	0.38^***^	−0.18^***^	0.28^***^	0.01	−		
11	ERQ	Suppression	4.00	1.03	0.78	−0.12^**^	−0.06	0.30^***^	−0.11^*^	0.17^***^	0.36^***^	−0.11^*^	0.04	0.03	0.52^***^	–	
12		Effortful control	2.67	0.35	0.88	−0.44^***^	0.23^***^	0.13^**^	−0.11^*^	0.08^†^	0.28^***^	0.22^***^	0.41^***^	0.58^***^	0.19^***^	0.16^***^	–
13		Toronto alexithymia 20	2.79	0.46	0.79	0.44^***^	−0.28^***^	−0.09^*^	0.08^†^	0.03	−0.13^**^	−0.33^***^	−0.47^***^	−0.48^***^	−0.10^*^	0.10^*^	−0.50^***^

a *Two items from Perspective Taking were excluded. IRI, Interpersonal Reactivity Index; FFMQ, Five Facets Mindfulness Questionnaire; ERQ, Emotion Regulation Questionnaire;*

*** *p < 0.001;*

** *p < 0.01;*

* *p < 0.05;*

† *p < 0.10*.

The Emotion Regulation Questionnaire (ERQ) is a 10-item scale and is constructed by two subscales [(68); Japanese version: (69)]: reappraisal (six items; α =0.84; Table 2) and suppression (four items; α = 0.78). We used the average of each subscale to represent these variables in the analyses. The participants responded on a seven-point scale (1: *strongly disagree*; 7: *strongly agree*).

The Effortful Control scale is a 35-item scale and is constructed by three subscales [(70); Japanese version: (71)]: inhibitory control (11 items), activation control (12 items), and attentional control (12 items). Correlations among the three subscales were high [(71): *r*s > 0.44; the present study: *r*s > 0.46), and the difference in mediation effects among them were beyond the focus of the present study. Therefore, the mean total of items was used for analyses, in order to assess broader cognitive control of attention and behavior (Table 2; α = 0.88). The participants responded on a four-point scale (1: *strongly disagree*; 4: *strongly agree*).

The Toronto Alexithymia Scale 20 (TAS 20) is a 20-item scale and is constructed by three subscales [(72); Japanese version: (73)]: difficulty identifying feelings (seven items), difficulty describing feelings (five items), and externally oriented thinking (eight items). The mean total of all items were used for analysis (Table 2; α = 0.79). The participants responded on a five-point scale (1: *strongly disagree*; 5: *strongly agree*).

#### Data Analyses

We conducted similar analyses to those conducted in Study 1. Additionally, for our main analysis, a mediation model (Supplementary Figure 1) was estimated by path analysis using Mplus [ver. 7.4; (31)]. In this model, the five scales of the FFMQ were entered as independent variables, and all their correlations were considered. Two scales of the ERQ (reappraisal and suppression), effortful control, and TAS20 were entered as mediation variables, and all correlations of their errors were considered. *empathic concern* and *perspective taking* of the IRI were entered as dependent variables, and the correlation of their errors was considered. Paths from the five scales of the FFMQ to the two scales of IRI and to all mediation variables were included in the model. Additionally, paths from all mediation variables to the two scales of the IRI were also considered in the model. Therefore, this mediation model was a saturated model. The bootstrapping method was conducted to estimate bias-corrected 95% confidence intervals (CIs) of indirect effects. The number of resampling was 2000.

As we did in Study 1, to investigate possible effects of demographic variables (gender and age), we included them in the path model (see Supplementary Material). Again, main results were not changed after adding these variables.

Additionally, with regard to mediation analyses, we added demographic variables (gender and age) to the abovementioned mediation model (see Supplementary Material). We assumed these demographic variables correlated with trait mindfulness (two-way path), all mediation variables (one-way path), and all dependent variables (one-way path). However, we did not assume a correlation between demographic variables.

Finally, we conducted all of Study 2's analyses after excluding participants who reported having meditation experience (*n* = 48; see Supplementary Material).

### Results

Results of correlation analyses are summarized in Table 2. *Observing* negatively and significantly correlated with *non-judging* and *acting with awareness* (*r*s < −0.30, *p* < 0.001), consistent with Study 1. *Non-reactivity* negatively and significantly correlated with *non-judging* (*r* = −0.21, *p* < 0.001), but did not significantly correlate with *acting with awareness* (*r* = −0.08, *p* = 0.088). Other internal correlations among the FFMQ were positive and significant (*r*s > 0.24, *p* < 0.001), except a non-significant negative correlation between *describing/labeling* and *non-judging* (*r* = −0.05, *p* = 0.273). Therefore, all results regarding internal correlations among the FFMQ are consistent with results of Study 1 except the correlation between *non-reactivity* and *acting with awareness*.

The estimated direct effects from the FFMQ to *empathic concern* and *perspective taking* were slightly different from the results of Study 1 (Figure 1). Inconsistent with Study 1, *non-reactivity* significantly and negatively correlated with *empathic concern* (β = −0.20, *p* < 0.001). Furthermore, paths from *non-judging* and *describing/labeling* to *empathic concern* and *perspective taking* were non-significant (βs < 0.11, *p* > 0.057). However, *observing* and *acting with awareness* significantly and positively correlated with *empathic concern* and *perspective taking* (βs > 0.15, *p* < 0.006), consistent with Study 1.

**Figure 2:**
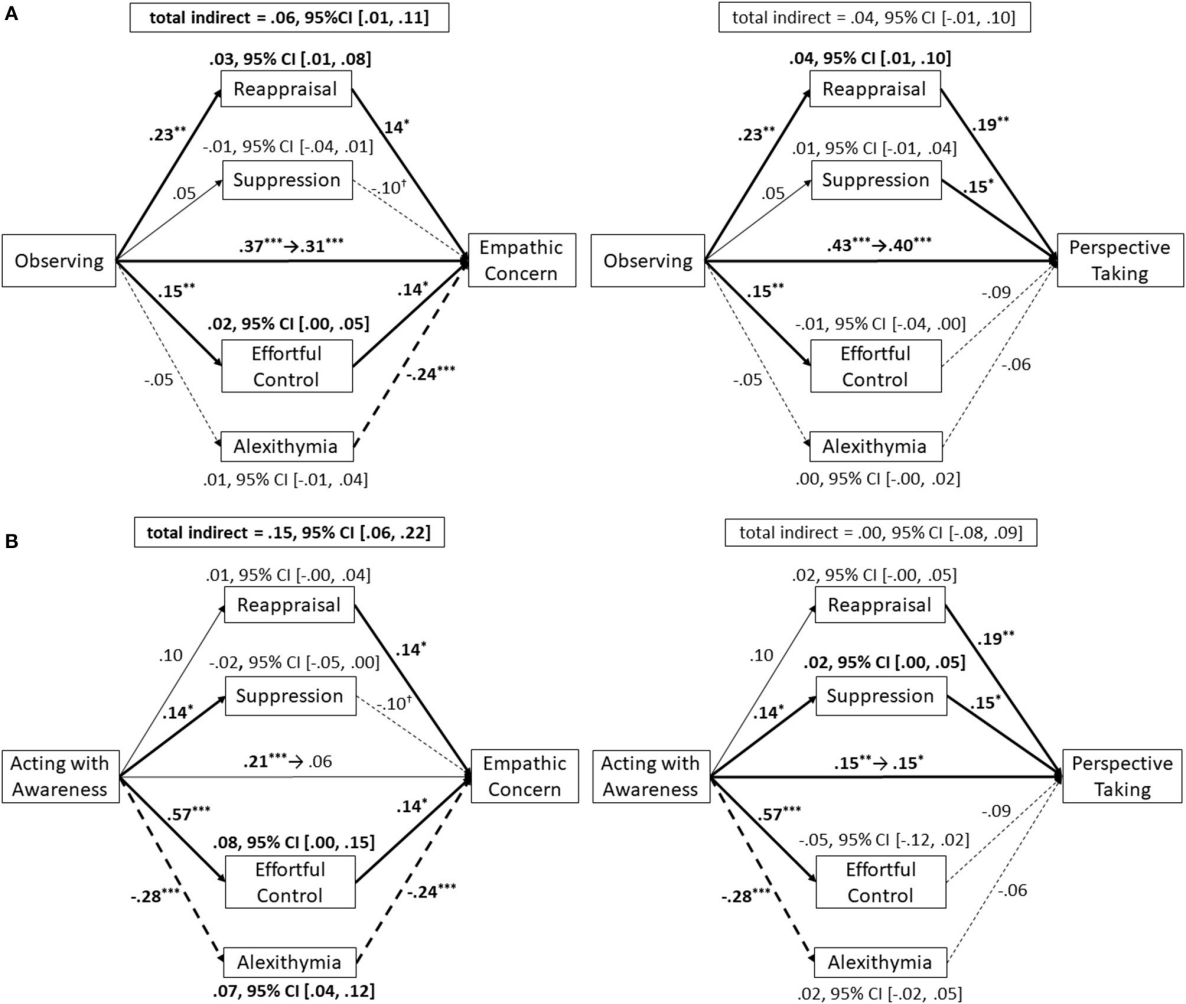
Results of path models for Studies 1 and 2 regarding the effects of the observing facet **(A)** and acting with awareness facet **(B)** on empathic concern (left) and perspective taking (right) through mediation variables. It should be noted that although this figure illustrates four intermediation models between facets of trait mindfulness and empathy, path analysis for each model conducted within the full model (Supplementary Figure 1). Each effect was standardized. Regarding direct effects, the left value on the center arrow indicates the effect without mediators and the right value of the arrow indicates effects after adding the mediators. ***p < 0.001; **p < 0.01; *p < 0.05; ^†^*p* < 0.10; 95% CI = 95% confidential interval.

As illustrated in Supplementary Figure 1, all mediation relationships were estimated simultaneously. However, we only report results regarding mediation relationships in which consistent, significant direct effects were found in both Studies 1 and 2. Correlations between errors of mediator variables and errors of dependent variables are illustrated in the (Supplementary Table 2). A total indirect effect of four mediation variables was found to be significant for helping explain the relationship between *observing* and *empathic concern* (Figure 2A; standardized total indirect effect = 0.06, 95% CI [0.01, 0.11]). After entering the mediation variables, a relationship between *observing* and *empathic concern* was decreased, but remained significant (β = 0.31, *p* < 0.001). Focusing on each simple indirect effect, reappraisal and effortful control significantly mediated the relationship between *observing* and *empathic concern* (reappraisal: standardized indirect effect = 0.03, 95% CI [0.01, 0.08]; effortful control: standardized indirect effect = 0.02, 95% CI [0.00, 0.05]). Although the total indirect effect from *observing* to *perspective taking* was not significant (Figure 2A; standardized total indirect effect = 0.04, 95% CI [−0.01, 0.10]), there was a simple indirect effect from *observing* to *perspective taking via* reappraisal (standardized indirect effect = 0.04, 95% CI [0.01, 0.10]) that partially mediated, and thereby decreased, the relationship (β = 0.40, *p* < 0.001).

A total indirect effect was significant for helping to explain the relationship between *acting with awareness* and *empathic concern* (Figure 2B; standardized total indirect effect = 0.15, 95% CI [0.06, 0.22]). A relationship between *acting with awareness* and *empathic concern* was non-significant after we entered the mediation variables (β = 0.06, *p* = 0.348). As regards this relationship, effortful control and TAS 20 were significant mediators (effortful control: standardized indirect effect = 0.08, 95% CI [0.00, 0.15]; TAS20: standardized indirect effect = 0.07, 95% CI [0.04, 0.12]). As regards the relationship from *acting with awareness* and *perspective taking*, the total indirect effect was non-significant (Figure 2B; standardized total indirect effect = 0.00, 95% CI [−0.08, 0.09]). In this relationship, although statistically non-significant, the negative indirect effect of effortful control was relatively large (standardized total indirect effect = −0.05, 95% CI [−0.12, 0.02]). Therefore, this negative indirect effect might cancel out other positive indirect effects.

After adding demographic variables, coefficients of *effortful control* on *empathic concern* slightly decreased and became non-significant (changed from β = 0.14, *p* = 0.039 to β = 0.11, *p* = 0.090). These results were also found after excluding the participants who had experienced meditation (changed from β = 0.14, *p* = 0.039 to β = 0.12, *p* = 0.086). As these changed, indirect effects of effortful control slightly decreased and became non-significant, both after adding demographic variables (Supplementary Figures 3A,B: *observing* → *empathic concern*: standardized indirect effect = 0.02, 95% CI [0.00, 0.04]; *acting with awareness* → *empathic concern*: standardized indirect effect = 0.06, 95% CI [−0.01, 0.13]) and after excluding the participants who had experienced meditation (Supplementary Figures 5A,B: *observing* → *empathic concern*: standardized indirect effect = 0.02, 95% CI [0.00, 0.05]; *acting with awareness* → *empathic concern*: standardized indirect effect = 0.07, 95% CI [−0.01, 0.15]). Because the coefficients changed slightly, moderation effects of these variables are small but present. Details of these results in are explained in Supplementary Material.

### Discussion

Using an independent sample, we investigated the reproducibility of relationships between trait mindfulness and trait empathy that were found in Study 1. Again, our path analysis results showed positive relationships between *observing* and the two primary subcomponents of empathy, and between *acting with awareness* and the two primary subcomponents of trait empathy (Figure 1). Nevertheless, the positive relationship between *describing/labeling* and *empathic concern* and the negative relationships between *non-judging* and the two subcomponents of empathy were not replicated. Therefore, our results suggest that traits that relate to the *attentional control* components of mindfulness robustly predict *empathic concern* and *perspective taking*, whereas the remaining relationships are non-significant or demonstrate low robustness. Our results support findings of previous studies (14, 26), insofar as *observing* and *acting with awareness* are positively correlated with trait empathy. Furthermore, positive relationships between *observing* and both emotional and cognitive components of empathy are consistent with the findings of another recent study (15). However, inconsistencies remain between the present study and previous studies, as described above. *Describing/labeling* was positively related to *perspective taking* in these previous studies, whereas these relationships were not significant in the present study. As described in discussion section of Study 1, cultural differences in interoception (43, 44) might have caused such inconsistency.

Study 2 showed that the relationship between *observing* and *empathic concern* is partially mediated by reappraisal and effortful control—although the effect effortful control was small and became statistically non-significant in our mediation analyses that included demographic variables and controlled for previous meditation experience (see Supplementary Material for more details). Nevertheless, previous studies have found that compassion training and emotion regulation strategies based on this training enhanced positive emotions and inhibited negative emotion for people in distressed situations (53, 54). Because the effects of meditation training on empathy may in part redound to changes in the ability to control one's emotions, our finding that reappraisal—and to an extent, effortful control—partially mediates the relationship between *observing* and *empathic concern* is consistent with previous results.

Intriguingly, Engen and Singer (53) found that emotion regulation based on compassion-meditation was more effective for enhancing positive emotions than emotion regulation based on reappraisal, whereas reappraisal was more effective at inhibiting negative emotions than a compassion-meditation-based strategy. Moreover, based on findings of neural responses of empathy toward others' distress, Weng et al. (12) suggested that increasing emotion regulation *via* meditation training may enhance positive emotions for people in the distressed situation, whereas reappraisal training may inhibit positive emotions for those people. These results suggest that the effects of meditation and reappraisal training on empathy may be different. Weng et al. (12) suggest that the inconsistency of the effects of compassion training and reappraisal training may be explained by the divergent goals of these training methods (compassion training: decreasing others' suffering; reappraisal training: decreasing one's own negative emotions). Considering these findings, although our results revealed that emotion regulation and cognitive control mediate the positive relationship between *observing* and *empathic concern*, the mere increase of emotion regulation could cause a negative outcome in some cases. Therefore, an important motivation factor (orientation toward one's own vs. another's suffering) may moderate our mediation model.

In a previous study, the suppression-based emotion regulation strategy, but not reappraisal, positively mediated relationships between *non-reactivity* and *empathic concern* and negatively mediated relationships between *describing/labeling* and *empathic concern* (14). However, in the present study, we did not focus on these relationships, because direct relationships between these two mindfulness components (i.e., *non-reactivity* and *describing/labeling*) and *empathic concern* were neither robust nor significant in our samples.

Study 2 showed that effortful control and alexithymia mediated relationships between *acting with awareness* and *empathic concern*—although the effect effortful control was small and became statistically non-significant in our mediation analyses that included demographic variables and controlled for previous meditation experience (see Supplementary Material for more details). The results regarding alexithymia are consistent with our predictions, but not regarding effortful control. Similar with the relationship between *observing* and *empathic concern*, results about effortful control are consistent with previous findings (12, 53). Regarding alexithymia, the function of the insula may be important for an interpretation of our results. The insula is associated with sharing other's emotions (62, 63) and helping behaviors (74, 75). A meta-analysis of neuroimaging studies about meditation has shown that training in various kinds of meditation increases insula activity (64). Additionally, a previous fMRI study has shown that activations of the anterior insula during observation of a signal of others' pain negatively correlates with the alexithymia tendency (61). These lines of evidence may support our mediation model.

However, at least one previous study has found that alexithymia did not mediate the relationship between any subcomponents of trait mindfulness and emotional empathy (15). Such inconsistency may be caused due to a conceptual discrepancy: emotional empathy measured in MacDonald and Price (15) focused on the sharing of another person's emotional state irrespective of whether one actually cared for the well-being of others (i.e., as in *empathic concern*). Taken together, our results suggest that the trait of focusing attention on one's own behavior, among the mindfulness subcomponents, positively relates with feeling compassion for others *via* a positive relationship with effortful control and a negative relationship with trait alexithymia.

Inconsistent with our predictions, the indirect effect of effortful control did not produce a significant relationship between either *observing* or *acting with awareness* and *perspective taking* (i.e., cognitive empathy). Apperly and Butterfill (56) point out that effortful control facilitates the reading of others' minds through an inhibition of information about one's own perspective. Consistent with this suggestion, psychological studies showed that cognitive load enhances egocentric bias [e.g., (58, 59)]. Therefore, our results are inconsistent with these lines of evidence. More studies using various measurements of cognitive control (e.g., performance on the n-back task or Stroop task) are needed to understand relationships among trait mindfulness, cognitive control, and *perspective taking*. Additionally, such investigations might contribute to checking the robustness of the relationship between effortful control and *empathic concern*, which demonstrated weak robustness in the present study (see Supplementary Material).

## General Discussion

Meditation training entails various cognitive and emotional changes such as the generation of compassion as well as mindfulness-related processes such as improvements in the self-monitoring of internal and external experiences, and the ability to withhold judgment as to whether experiences are good or bad (16). The variety of processes underlying meditation training and related changes in mindfulness, however, may cause ambiguity as to the mechanisms that explain various outcomes, including increases in empathy. Narrowing our investigation to the trait level, the results of the present study suggests that two attentional-related components of trait mindfulness—*observing* and *acting with awareness*—are important for enhancing *empathic concern* on the one hand and *perspective taking* on the other. These positive relationships were also found in previous studies conducted in Western cultures (14, 26); therefore, these relationships may be robust across cultures. Additionally, we found that effortful control is a common mediation factor in the relationships between the two attentional components of trait mindfulness and emotional empathy (but its weak robustness should be noted; see Supplementary Material), whereas reappraisal and trait alexithymia are specific mediation factors in relationships between emotional empathy and *observing* and between emotional empathy and *acting with awareness*, respectively. These relationships are partly consistent with previous experimental findings (12).

Because our studies investigated trait mindfulness and trait empathy, we cannot fully speak to the relationship between state-level mindfulness (a concomitant of meditation) and state-level empathy. Nevertheless, our results imply that a specified training focusing on cognitive—attentional—components of meditation may be important for both cognitive and emotional empathy. These relationships are perhaps most intriguing in terms of their effects on emotional empathy: two attention-related components of mindfulness, which in and of themselves have no obvious relationship to emotionality, are nonetheless associated with medium effects on emotional empathy. Understanding exactly why people who are high in the ability of *observing* (mindful observation) and who have a tendency for *acting with awareness* (mindful action) show higher levels of *empathic concern* presents an interesting direction for future research.

## Limitations and Future Research

There are several limitations in the present study. The first, as mentioned before, is that our studies did not manipulate meditation; instead, we investigated relationships among trait mindfulness, mediation factors (only psychological traits), and trait empathy. Therefore, it should be noted that the present mediation relationships are not causal. Second, data of the present study were collected with the cross-sectional method; thus, it should be noted that temporal sequences of the path models in the present study cannot be established. Future studies should investigate whether our model is supported in the context of meditation training interventions, in order to overcome limitations about causality and temporal sequences. Third, we used self-reports to measure mindfulness, empathy, and mediation variables, and thus, it should be noted that such measurements only indirectly assess these constructs. Fourth, our participants were mostly naive to the practice of meditation. Previous studies have shown that correlations between *observing* and psychological well-being and between *observing* and several psychological outcomes are different depending on the length of meditation experience (21, 22). Therefore, whether our results may be applied to people who are currently practicing meditation is still unclear. Meta-analysis has suggested that meditation increases activities of the insula (64). The insula plays an important role in sharing other's emotions (62, 63, 76) and interoception (77). Therefore, components of mindfulness associated with bodily sensations might relate to emotional empathy more strongly in people trained in meditation than novices. Fifth, although the FFMQ gives a relatively comprehensive measurement of trait mindfulness, it does not mean that this measurement can completely capture all aspects of mindfulness. For example, socio-cognitive mindfulness is not measured by the FFMQ, and a previous study has shown that this component of trait mindfulness positively related to emotional and cognitive empathy (78).

The relationships between trait mindfulness and trait empathy are partly inconsistent between the present study and previous studies (14, 15, 26). Specifically, *describing/labeling* positively related to *perspective taking* in the previous studies, whereas this scale related to *empathic concern*, but with relatively low robustness. As described above, cultural difference in interoception (43, 44) might be one of the factors which caused these inconsistencies. Comparing the relationship between mindfulness and empathy (and mediation factors) with different cultural samples while using the same scales and behavioral paradigms (e.g., heartbeat detection task) would allow researchers to investigate this possibility. Such investigations are important for understanding the moderation effects of macro-factors (e.g., social norms and cultural self-construal) on meditation/mindfulness and empathy.

## Conclusions

Although previous studies have shown a positive relationship between mindfulness (usually as a result of meditation) and empathy, their relationship is complex, occurring at both the state and trait levels. Furthermore, trait mindfulness and trait empathy are constructed of various subcomponents, and meditation is known to affect these and other psychological variables. Focusing on the trait level, our study found that attention-related components of mindfulness reliably correlate with not only cognitive empathy but also emotional empathy, and several emotional and cognitive traits mediate relationships between attentional components of mindfulness and emotional empathy. Future studies should focus on detailing relationships between mindfulness and empathy, for example examining the specific sub-processes in mindfulness practice that efficiently influence emotional or cognitive empathy. Such an approach may contribute to a mechanistic understanding of meditation's effect on empathy. We hope that the present study with its focus on the multidimensional nature of the links between mindfulness and empathy at the trait level will serve as a foundation for future research.

## Data Availability Statement

The raw data supporting the conclusions of this article will be made available by the authors, without undue reservation.

## Ethics Statement

The studies involving human participants were reviewed and approved retrospectively by Department of Cognitive Psychology in Education from Kyoto University. Written informed consent for participation was not required for this study in accordance with the national legislation and the institutional requirements.

## Author Contributions

TH performed nearly all processes of Studies 1 and 2 (development of the study concept, study design, and data analysis) and wrote the manuscript. HO, TG, HF, and YK contributed to development of the study concept and the study design for Study 1 and provided the critical revisions for the manuscript. AS wrote the manuscript and provided critical revisions. MN supervised nearly all processes of Studies 1 and 2 (development of the study concept, study design, and data analysis) and provided critical revisions for the manuscript. All the authors approved the final version of the manuscript.

## Conflict of Interest

The authors declare that the research was conducted in the absence of any commercial or financial relationships that could be construed as a potential conflict of interest.

## Publisher's Note

All claims expressed in this article are solely those of the authors and do not necessarily represent those of their affiliated organizations, or those of the publisher, the editors and the reviewers. Any product that may be evaluated in this article, or claim that may be made by its manufacturer, is not guaranteed or endorsed by the publisher.
