# Supplementary material for: Exploring the Multidimensional Links Between Trait Mindfulness and Trait Empathy
Source: Front Psychiatry. 2021 Aug 4;12:498614. doi: 10.3389/fpsyt.2021.498614 (PMC8371256; doi:10.3389/fpsyt.2021.498614)
Supplement: Supplementary file 1 [file Data_Sheet_1.docx]

Supplementary Materials

**1. Path model from Studies 1 and 2 regarding the direct relationship between trait mindfulness and trait empathy after adding demographic variables**

We investigated the moderation effects of demographic variables (i.e., gender and age). In specific, we added age and a dummy variable of gender (1: men; 2: women) to our path models (from Study 1 and Study 2) as control variables.Results are illustrated in Figure S2. Model fitness was good in Study 1 and Study 2 (Study 1: χ2 (1) = 0.02, *p* = .892; CFI = 1.000, RMSEA < .001; Study 2: χ2 (1) = 0.07, *p* = .799; CFI = 1.000, RMSEA < .001). Consistent with the main analyses of the two studies, the direction and statistical significance levels (i.e., *p*< .001,*p*< .01, *p*< .05, and *p*< .10) of almost correlations and standardized coefficients were identical to those of Study 1 and Study 2. The size of several effects did, however, vary; but this variance was never greater than *r* = .03 and followed no obvious pattern. Therefore,meaningful moderation effects of demographic variables were not found.

**2. Mediation model from Study 2 after adding demographic variables**

We conducted our mediation analyses after adding demographic variables (i.e., gender and age), assuming these demographic variables correlated with trait mindfulness (two-way path), all mediation variables (one-way path), and all dependent variables (one-way path). As mentioned in the main text, we did not assume a correlation between demographic variables.

Model fitness after adding demographic variables (i.e., gender and age) to the mediation modelwas good (χ^2^ (1) = 0.07, *p* = .799; CFI = 1.000, RMSEA < .001). Results are illustrated in Figure S3. Unlike our main analysis, relationships of *effortful control* on *empathic concern* become non-significant (β = .11, *p* = .090). As a result,in the relationship between *observing* and *empathic concern*, the indirect effect of effortful control was nonsignificant (standardized indirect effect = .02, 95% CI [.00, .04]), despite no change in its magnitude. In the relationship between *acting with awareness* and *empathic concern*, the indirect effect of effortful control was likewise nonsignificant (standardized indirect effect = .06, 95% CI [-.01, .13]). These results suggest that moderation effects of demographic variables, as related to effortful control, though small, are present.

Other than the abovementioned changes, the effects and significance patterns of the mediation model were unaffected by the addition of demographic variables.

**3. Data analyses from Study 2 including only participants without meditation experience**

We conducted a set of data analyses based onStudy 2 in the main text using a dataset which excluded participants who reported to have meditation experience (*n* = 48). Analyzing only participants without meditation experience, four hundred sixty-eight participants were included in this dataset (229 men and 239 women; mean age = 39.25, standard deviation [*SD*] = 11.05).

Basic statistical values for each scale and correlation analyses aresummarized in Table S3. Results wereequivalent with results of the main analyses (Table 2).In other words, the direction and statistical significance levels (i.e., *p*< .001, *p* < .01, *p* < .05, and *p*< .10) of nearly all correlations were identical to those of Study 2. The size of most correlation’s didvary; but this variance was never greater than *r* = .04 except for the following correlations: *perspective taking*and*alexithymia* changed from *r* = -.09, *p*< .05 to *r* = -.15, *p*< .01; *acting with awareness* and *nonreactivity* changed from *r* = -.08, *p*< .10 to *r* = -.03, *p* = .484; and *nonreactivity* and *alexithymia* changed from *r* = -.13, *p*< .01 to *r* = -.18, *p*< .001. In short, no meaningful changes to the bivariate correlation patterns were found.

We next estimated direct effects from each component of the FFMQ to *empathic concern* and *perspective taking*fromthe IRI*,*and indirect effects of mediation model in Study 2. Results of the former analyses are illustrated in Figure S4, and these results are entirely consistent with the main analysis (Figure 1; Study 2).The direction and statistical significance levels (i.e., *p*< .001, *p*< .01, *p*< .05, and *p*< .10) of almost correlations and standardized coefficients were identical to those of Study 1 and Study 2. The size of some effects did vary, but this variance was typically small and never exceeded *r* = .05. Once again and central to the present study, *observing*and *acting with awareness* positively and significantly correlated with *empathic concern* and *perspective taking*(βs > .16, *p*-values < .006).

Regarding mediation analyses, we focused on indirect effects associated with relationships among two components of the FFMQ (i.e., *observing* and *acting with awareness*)and two components of the IRI (i.e., *empathic concern* and *perspective taking*) which demonstrated significant direct effects in the abovementionedpath model. Results of these analyses are illustrated in Figure S5. Dissimilar with the results of Study 2 (Figure 2), the relationships between effortful control and *empathic concern* were nonsignificant (β = .12, *p* = .086); thus indirect effects of effortful control werenonsignificant (Figure S5A and B: *observe*→ *empathic concern*: standardized indirect effect = .02, 95% CI [.00, .05]; *acting with awareness*→*empathic concern*: standardized indirect effect = .07, 95% CI [-.01, .15]). Otherwise, the mediation analyses, followed the same pattern of effects as the main analyses.

Even though almost results of analyses after excluding the participants who had meditation experiences were identical with the main results, it is noteworthy that the relationship between effortful control and empathic concern became statistically nonsignificant. A possible interpretation for these results is the moderation effects of meditation. However, recall that although forty-eight participants responded that they had meditation experience, only ten participants had experience of meditation practice greater than the required amount of time in standard intervention studies (twenty-four hours, in total). Therefore, an interpretation that assumes moderation effects of meditation, in the present study, may not be suitable. An alternative interpretation of these results is lowered power due to reduced sample size.

**Table S1.**

*Duration (in months) and time (in hours) of meditation practice for the participants from Study 2 who have meditation experience (n = 48)*

| Meditation practice duration | *n* |
| --- | --- |
| Less than 12 months | 26 |
| 13 - 24 months | 10 |
| 25 - 60 months | 7 |
| 61 - 120 months | 3 |
| 121 - 245 months | 2 |
| Meditation practice time | *n* |
| Less than 24 hours | 38 |
| 25 -50 hours | 3 |
| 51 -100 hours | 2 |
| 101 - 500 hours | 4 |
| 501 - 1000 hours | 1 |

**Table S2.**

*Correlations between errors of mediator variables and errors of dependent variables in the full mediation model*

|  |  |  |  | *r* | *p* |
| --- | --- | --- | --- | --- | --- |
| mediation variables | error Reappraisal | ⇔ | error Suppression | .46 | <.001 |
|  | error Reappraisal | ⇔ | error Effortful Control | .04 | .472 |
|  | error Reappraisal | ⇔ | error Alexithymia | .00 | .995 |
|  | error Suppression | ⇔ | error Effortful Control | .06 | .298 |
|  | error Suppression | ⇔ | error Alexithymia | .16 | .001 |
|  | error Effortful Control | ⇔ | error Alexithymia | -.20 | .001 |
| dependent variables | error Empathic Concern | ⇔ | error Perspective Taking | .35 | <.001 |

**Table S3**

*Basic statistical values, internal reliabilities, and correlations among each scale in Study 2 (participantswithout meditation experience)*

|  | scale |  | *M* | *SD* | α | 1 | 2 | 3 | 4 | 5 | 6 | 7 | 8 | 9 | 10 | 11 | 12 |
| --- | --- | --- | --- | --- | --- | --- | --- | --- | --- | --- | --- | --- | --- | --- | --- | --- | --- |
| 1 | IRI | Personal Distress | 3.08 | 0.62 | .74 | - |  |  |  |  |  |  |  |  |  |  |  |
| 2 | IRI | Empathic Concern | 3.31 | 0.59 | .77 | .04 | - |  |  |  |  |  |  |  |  |  |  |
| 3 | IRI | Perspective Taking^a^ | 3.02 | 0.65 | .75 | -.02 | .42^***^ | - |  |  |  |  |  |  |  |  |  |
| 4 | IRI | Fantasy Scale | 3.13 | 0.69 | .80 | .21^***^ | .31^***^ | .30^***^ | - |  |  |  |  |  |  |  |  |
| 5 | FFMQ | Observing | 2.81 | 0.63 | .79 | .03 | .25^***^ | .46^***^ | .31^***^ | - |  |  |  |  |  |  |  |
| 6 | FFMQ | Non-reactivity | 2.82 | 0.57 | .75 | -.35^***^ | .00 | .27^***^ | .01 | .42^***^ | - |  |  |  |  |  |  |
| 7 | FFMQ | Non-judging | 3.17 | 0.61 | .82 | -.26^***^ | -.06 | -.18^***^ | -.26^***^ | -.53^***^ | -.17^***^ | - |  |  |  |  |  |
| 8 | FFMQ | Describing/Labeling | 2.82 | 0.66 | .84 | -.37^***^ | .18^***^ | .25^***^ | .01 | .29^***^ | .38^***^ | -.04 | - |  |  |  |  |
| 9 | FFMQ | Acting with Awareness | 3.35 | 0.66 | .86 | -.41^***^ | .16^***^ | .04 | -.18^***^ | -.29^***^ | -.03 | .47^***^ | .27^***^ | - |  |  |  |
| 10 | ERQ | Reappraisal | 4.15 | 0.88 | .85 | -.13^**^ | .17^***^ | .43^***^ | .11^*^ | .33^***^ | .37^***^ | -.16^**^ | .26^***^ | .04 | - |  |  |
| 11 | ERQ | Suppression | 3.99 | 1.03 | .79 | -.13^**^ | -.05 | .28^***^ | -.12^**^ | .14^**^ | .33^***^ | -.07 | .02 | .05 | .50^***^ | - |  |
| 12 |  | Effortful Control | 2.67 | 0.36 | .88 | -.43^***^ | .24^***^ | .16^**^ | -.10^*^ | .09^*^ | .31^***^ | .21^***^ | .41^***^ | .60^***^ | .21^***^ | .18^***^ | - |
| 13 |  | Toronto Alexithymia 20 | 2.78 | 0.45 | .79 | .45^***^ | -.28^***^ | -.15^**^ | .05 | .01 | -.18^***^ | -.31^***^ | -.50^***^ | -.48^***^ | -.13^**^ | .06 | -.49^***^ |

*Notes.* a: Two items of Perspective Taking scale were excluded. IRI = Interpersonal Reactivity Index; FFMQ = Five Facets Mindfulness Questionnaire, ERQ = Emotion Regulation Questionnaire; ***: *p*< .001; **: *p*< .01; *: *p*< .05; †: *p*< .10


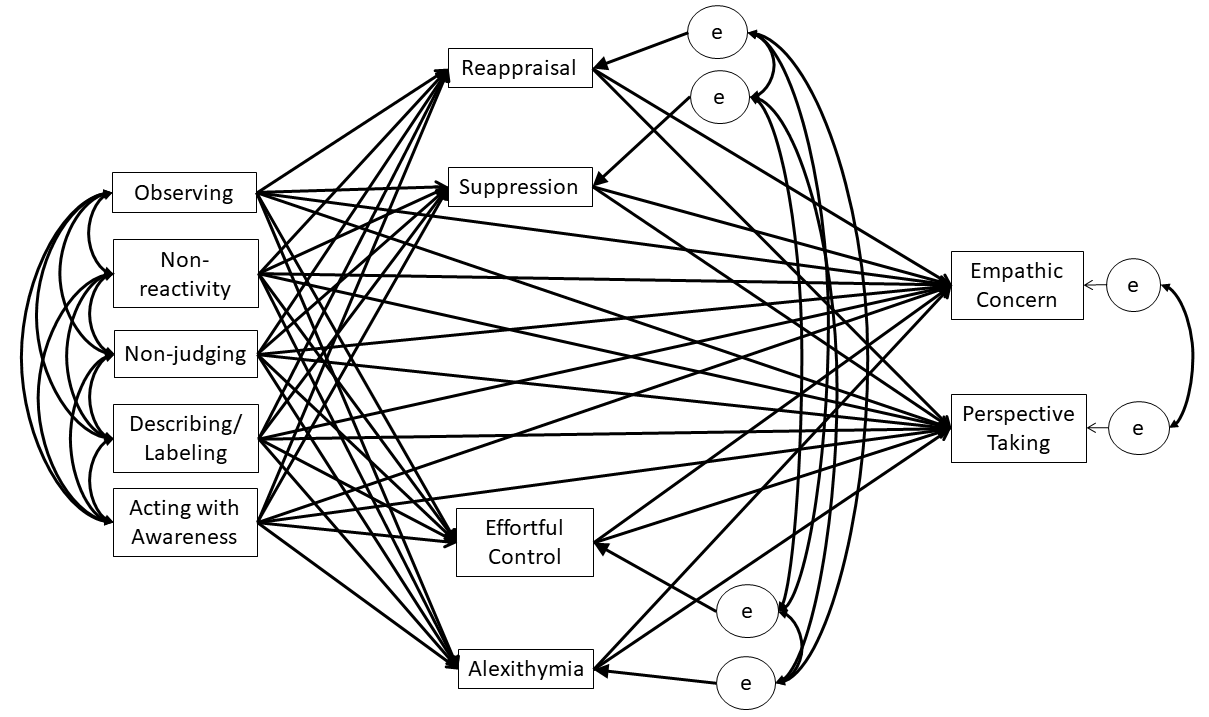


**Figure S1.Intermediation model which was investigated by path modeling.** e = error.


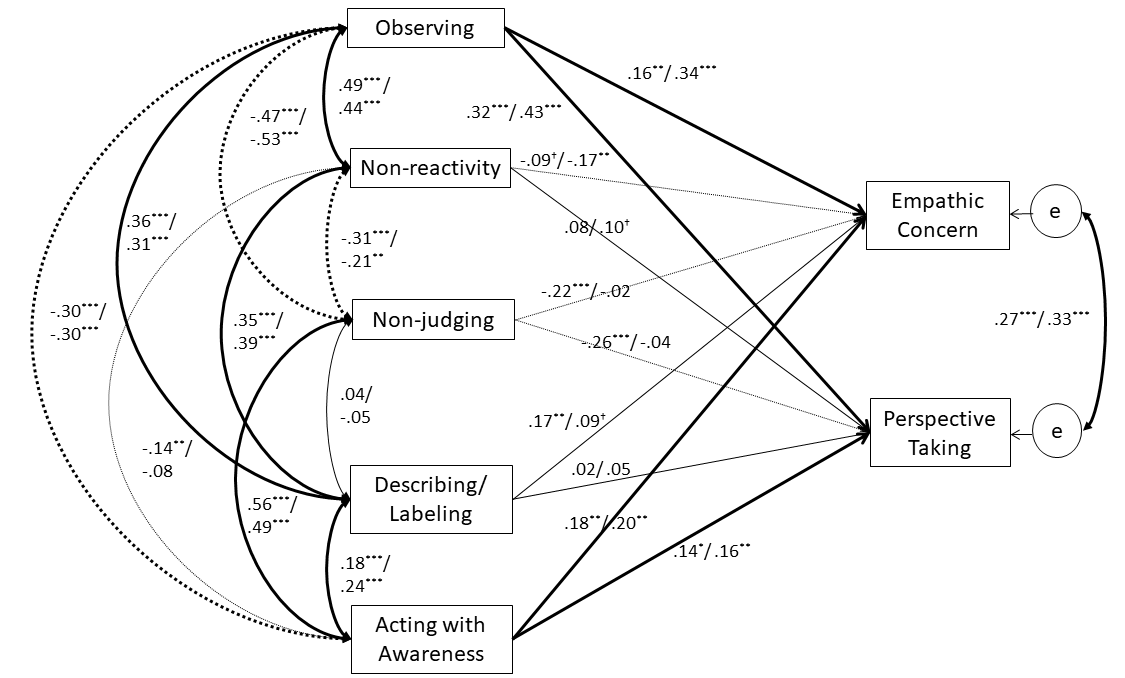


**Figure S2. Results of path models for Studies 1 and 2 regarding the effects of five facets of mindfulness on empathic concern and perspective taking after adding demographic variables**. Each value indicates a correlation (*r*) or standardized coefficient (β). Weights of paths correspond to statistical significance of each path, both in Study 1 and Study 2. Left values are results of the Study 1, and right values are results of Study 2. Continuous lines indicate positive relationships, and dotted lines indicate negative relationships. In these analyses, although demographic variables (i.e., gender and age) were included as control variables, these results were removed from this figure. ***: *p*< .001; **: *p*< .01; *: *p*< .05; †: *p*< .10; e = error.


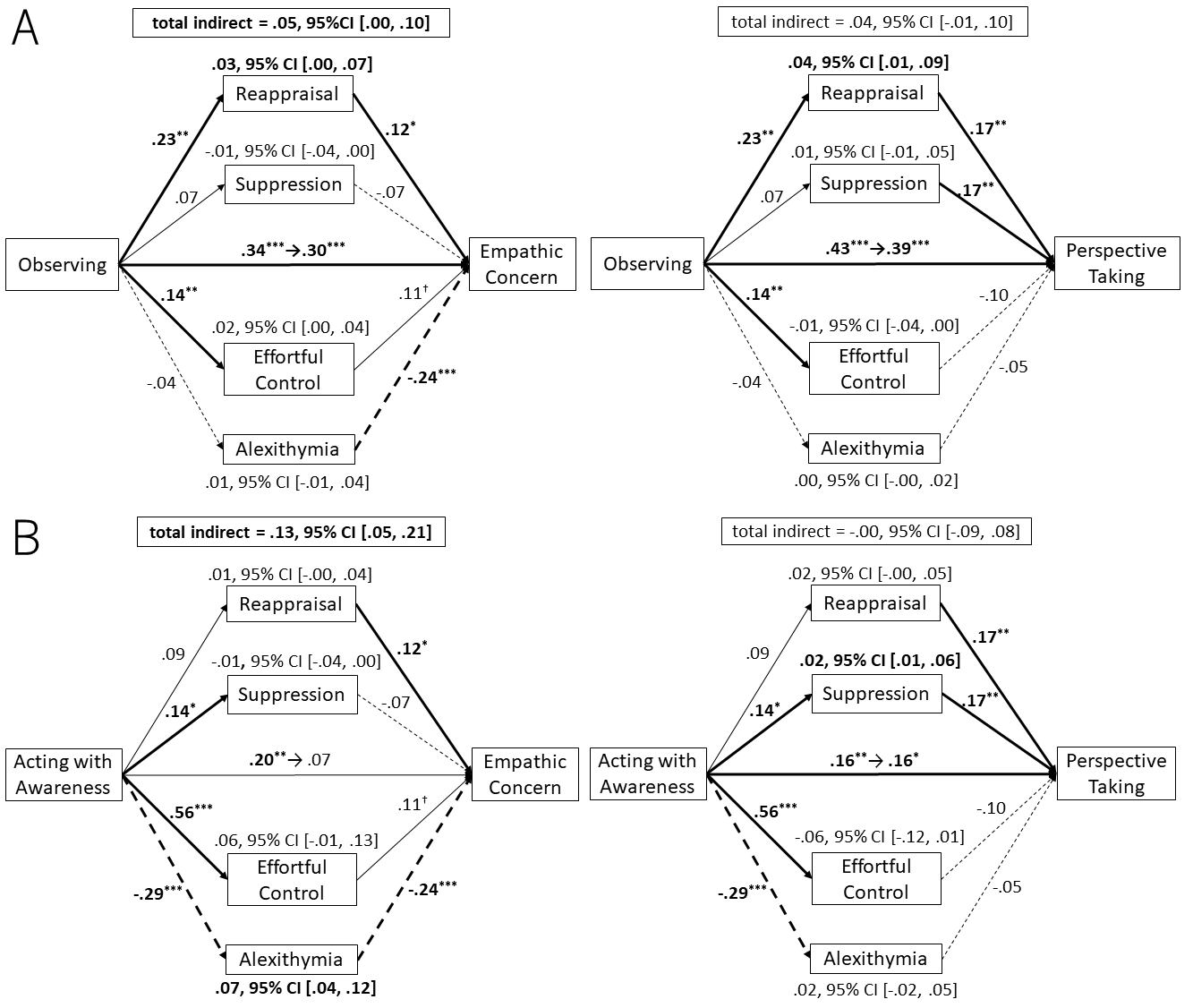


**Figure. S3. Results of path models regarding the effects of the *observing* facet (A) and *acting with awareness* facet (B) on *empathic concern* (left) and *perspective taking* (right) through mediation variables with demographic variables.** It should be noted that although this figure illustrates four intermediation models between facets of trait mindfulness and empathy, model testing for each model was conducted within the full model after adding demographic variables (i.e., gender and age). Each effectis standardized. Regarding direct effects, the left value of on the center arrow indicates the effect without mediators and the right value of the arrow indicates effects after adding the mediators. ***: *p*< .001; **: *p*< .01; *: *p*< .05; †: *p*< .10; 95%CI = 95% confidential interval.


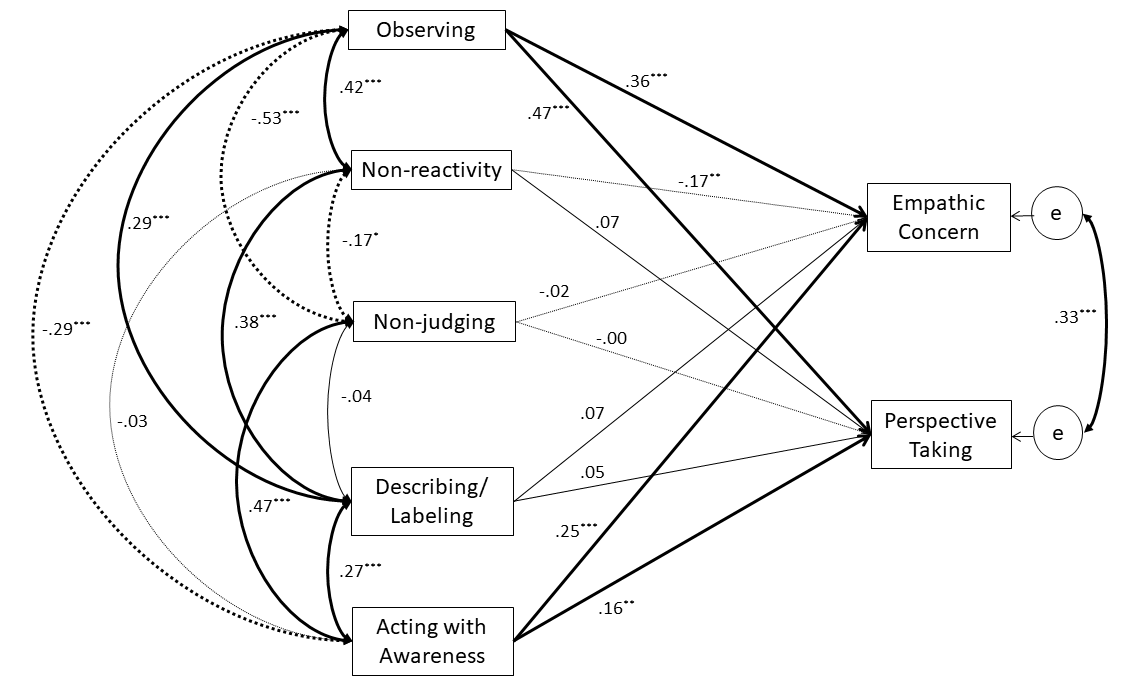


**Figure S4. Results of a path model regarding effects of the five facets of mindfulness on empathic concern and perspective taking in participants without meditation experience (*n* = 468)**. Each value indicates a correlation (*r*) or standardized coefficient (β). Weights of paths correspond to statistical significance of each path, both in Study 1 and Study 2. Continuous lines indicate positive relations, and dotted lines indicate negative relations. ***: *p*< .001; **: *p*< .01; *: *p*< .05; †: *p*< .10; e = error.

**
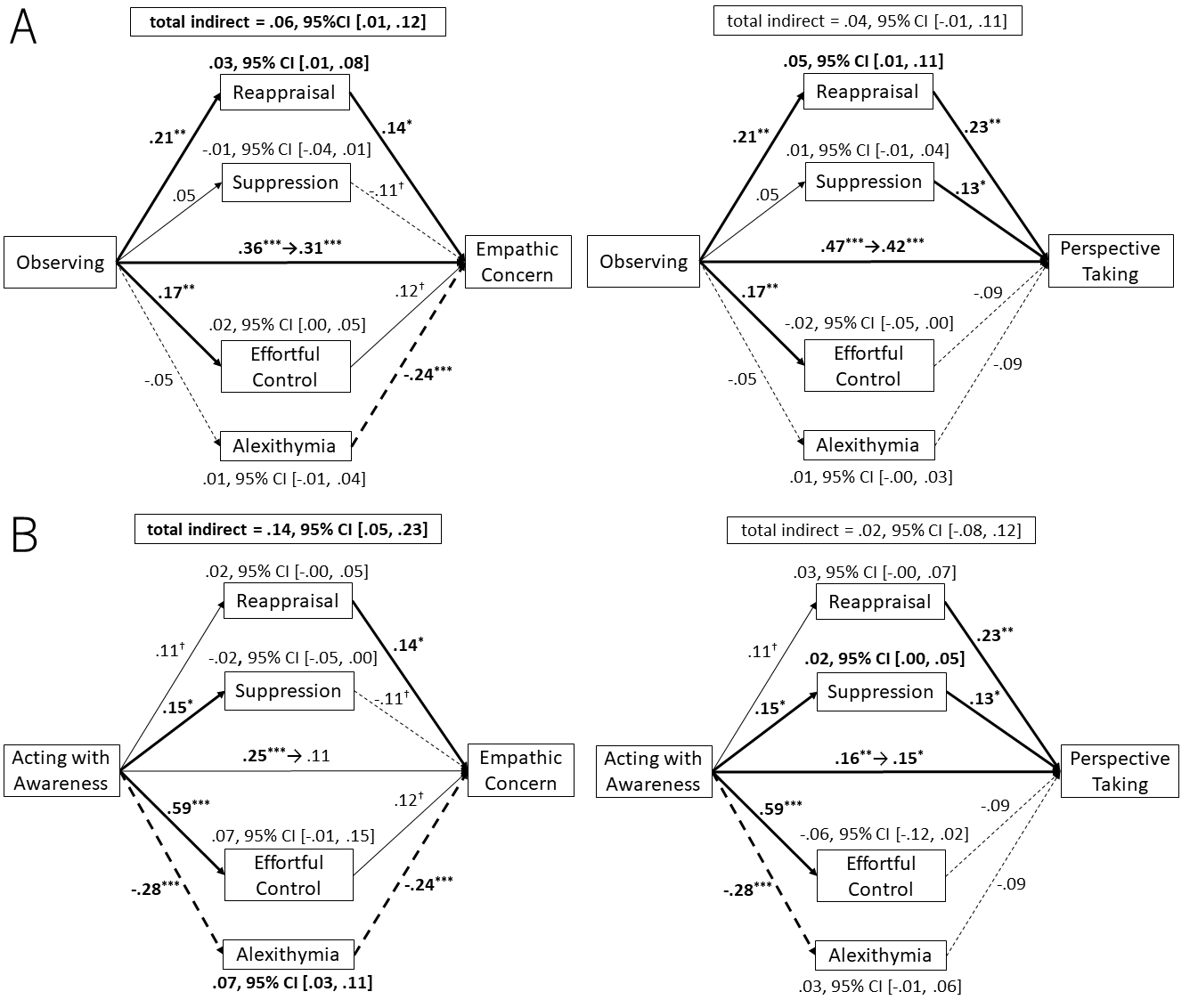
**

**Figure S5. Results of path models regarding the effects of the observing facet (A) and acting with awareness facet (B) of thee FFMQ on empathic concern (left) and perspective taking (right) through mediation variables in participants without meditation experience (*n* = 468).** It should be noted that although this figure illustrates four intermediation models between facets of trait mindfulness and empathy, model testing for each model was conducted within the full model (Figure S1). Each effect was standardized. Regarding direct effects, the left value of the center arrow indicates the effect without mediators and the right value indicates effects after adding the mediators. ***: *p*< .001; **: *p*< .01; *: *p*< .05; †: *p*< .10; 95%CI = 95% confidential interval.
